# Venetoclax as a cytoreduction therapy option for acute promyelocytic leukemia in newly diagnosed adult patients: a case report of a 35-year-old female with schizophrenia

**DOI:** 10.3389/fonc.2025.1671529

**Published:** 2025-09-18

**Authors:** Lingling Wang, Yuqing Miao, Yuexin Cheng

**Affiliations:** Department of Hematology, The First People’s Hospital of Yancheng, The Yancheng Clinical College of Xuzhou Medical University, Yancheng, Jiangsu, China

**Keywords:** acute promyelocytic leukemia, schizophrenia, cytoreduction, venetoclax, differentiation syndrome

## Abstract

Venetoclax is effective in treating relapsed acute promyelocytic leukemia (APL), newly diagnosed pediatric APL, variant APL, and APL with central nervous system involvement. In newly diagnosed adult APL, venetoclax is rarely used. Herein, we present a case of newly diagnosed adult APL in a 35-year-old female with schizophrenia who received venetoclax as a cytoreduction therapy option. The patient was admitted with myocardial ischemia, the cardiac ultrasound indicating left ventricular ejection fractions (EF) of 44%, a 17-year history of schizophrenia, treated with ziprasidone, lorazepam, and clozapine. She developed differentiation syndrome (DS) shortly after receiving All-trans-retinoic acid (ATRA) and arsenic trioxide (ATO) and experienced heart arrest. In the occurrence of DS, this young female encountered numerous therapeutic conundrums, including cytoreduction of hydroxyurea being ineffective, the potential psychological worsening caused by dexamethasone use, and the cardiotoxicity of anthracyclines. We administered venetoclax 20 mg once daily as a cytoreduction therapy. The white blood cells (WBC) dropped from 72.16×10^9^/L to 5.19×10^9^/L in 4 days, and the proportion of promyelocytes in the peripheral blood smear decreased from 78% to 10%. Tumor lysis syndrome (TLS) did not develop since the patient received good supportive treatment. For newly diagnosed adult patients with APL who are unresponsive to traditional cytoreduction therapy, venetoclax can be an effective option.

## Background

Acute promyelocytic leukemia (APL) is a special type of acute myeloid leukemia (AML) (1). More than 98% of patients with bone marrow morphology of APL have the typical t (15,17) chromosome translocation and *PML::RARα* fusion gene. APL often begins with severe bleeding as the first manifestation, and the onset of the disease can be very aggressive and progress rapidly, which can lead to early death if not treated in time (2). Patients with white blood cells (WBC) ≥ 10×10^9^/L are considered high-risk APL, which poses significant challenges for clinical management due to their poor clinical prognosis and high early mortality rate. With chemotherapy alone, the remission rate for classic APL was between 47% and 88%. With the advent of All-trans-retinoic acid (ATRA) (3), the 5-year overall survival (OS) rate can reach 80% to 90%, and the remission rate after treatment with ATRA + chemotherapy can surpass 90%. The treatment regimen utilizing ATRA + arsenic trioxide (ATO) ± anthracycline further decreased chemotherapy-related complications (4). The 5-year OS rate was over 90% (5–7). However, some patients still experience early mortality within 30 days of induction therapy due to severe complications. Since high tumor burden and elevated WBC are significant contributing factors, cytoreduction therapy must be applied judiciously to lower early mortality in APL. Conventional cytoreduction therapy does not work for all patients. Therefore, it is crucial to discover new cytoreduction drugs to reduce early mortality.

Venetoclax is an inhibitor targeting Bcl-2 (8). The combination of venetoclax with azacitidine and FLT-3 inhibitors synergistically enhances anti-leukemic activity (9). For newly diagnosed adult AML (non-APL), venetoclax combined with hypomethylating agents is used to treat patients who are unfit for intense induction chemotherapy. Venetoclax is effective in treating relapsed APL (10), newly diagnosed pediatric APL (11), variant APL (12–16), and APL with central nervous system involvement (17). It is anticipated that venetoclax, which has been used in several clinical studies as cytoreduction therapy (11) or even as first-line treatment for APL (e.g., ChiCTR2400085721), may reduce the incidence of disease-related complications, making it a superior option. In newly diagnosed adult APL, venetoclax is rarely used. Herein, we present a case of a 35-year-old female with schizophrenia who received venetoclax as a cytoreduction therapy.

## Case presentation

**Figure 1:**
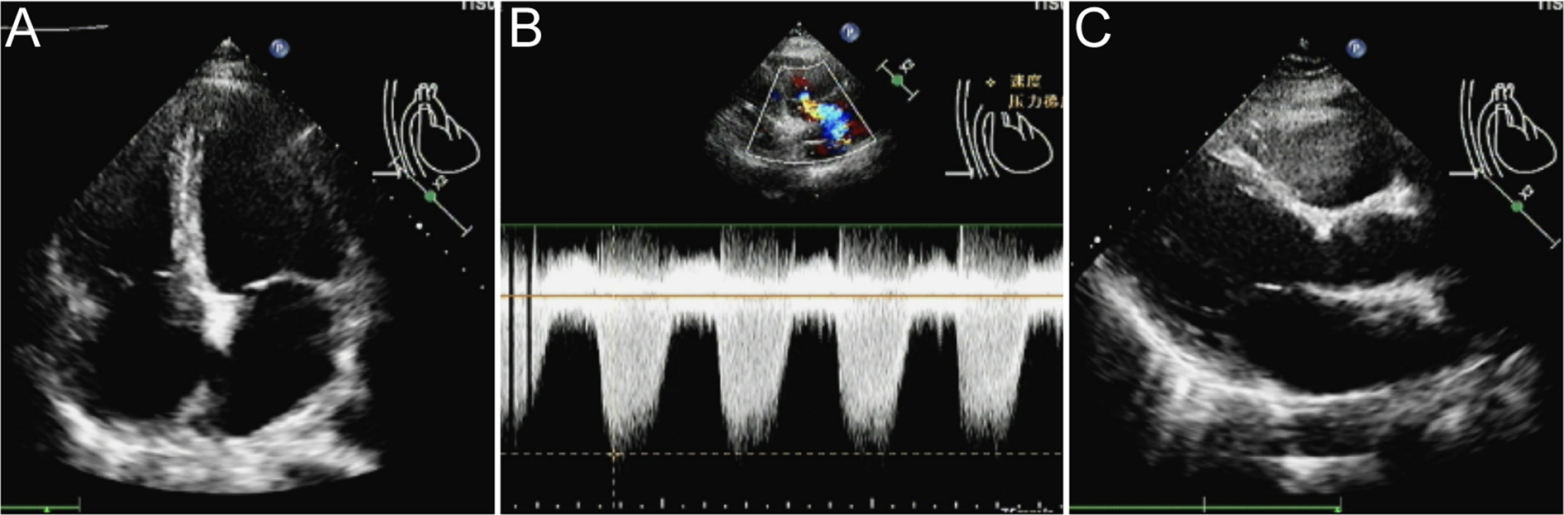
The cardiac ultrasonography. **(A)** The left atrium was enlarged, with mitral and tricuspid regurgitation. **(B, C)** The pulmonary artery systolic pressure was 40 mmHg, and the EF was only 44%.

A 35-year-old female was admitted to our emergency department at 3:00 a.m. on February 25, 2024. She complained of palpitations, dizziness, and widespread weakness, along with gingival and vaginal bleeding. The local hospital’s laboratory results indicated WBC 8.1×10^9^/L, hemoglobin 46 g/L, platelets 4×10^9^/L, prothrombin time (PT) 20.7 s (normal range 9-14.5 s), fibrinogen 1.11 g/L (normal range 2-4 g/L), D-dimer 33.39 mg/L (normal range 0-0.5 mg/L), lactate dehydrogenase (LDH) 907.0 U/L (normal range 120-250 U/L), and high-sensitivity cardiac troponin (hs-cTn) 35.4 ng/L (normal range <14 ng/L). The electrocardiogram (ECG) revealed sinus tachycardia, premature ventricular contractions, ST-segment depression, and T-wave anomalies, all of which pointed to anterior wall myocardial ischemia. The patient had a 17-year history of schizophrenia, treated with ziprasidone, lorazepam, and clozapine. On admission, her temperature was 38.4°C, pulse was 130 beats per minute, respiration rate was 21 beats per minute, and blood pressure was 134/74 mmHg. According to the patient’s cardiac ultrasonography, the pulmonary artery systolic pressure (PASP) was 40 mmHg, the left atrium was enlarged, mitral and tricuspid regurgitation, and the left ventricular ejection fraction (EF) was only 44% (normal range 50%-70%) (**Figure 1**). The proportion of promyelocytes in the peripheral blood smear was 92%. The patient received bone marrow aspiration on the day of admission. The smear showed premyelocytes accounted for 90%. A group of naïve cells that expressed CD33+CD117+CD13+CD64+CD14+CD15+CD56+CD38+ accounted for 91.1% on flow cytometry, and were negative for CD34 and HLA-DR. All the tests indicated APL. We immediately administered ATRA 10mg three times a day plus realgar-indigo naturalis formula (RIF) 1.35g three times a day to reduce the risk of disseminated intravascular coagulation (DIC). The patient’s diagnosis was verified by a follow-up chromosomal and fusion gene (46, XX, t (15,17) (q24;q21) [10], and *PML::RARα*, respectively).

**Figure 2:**
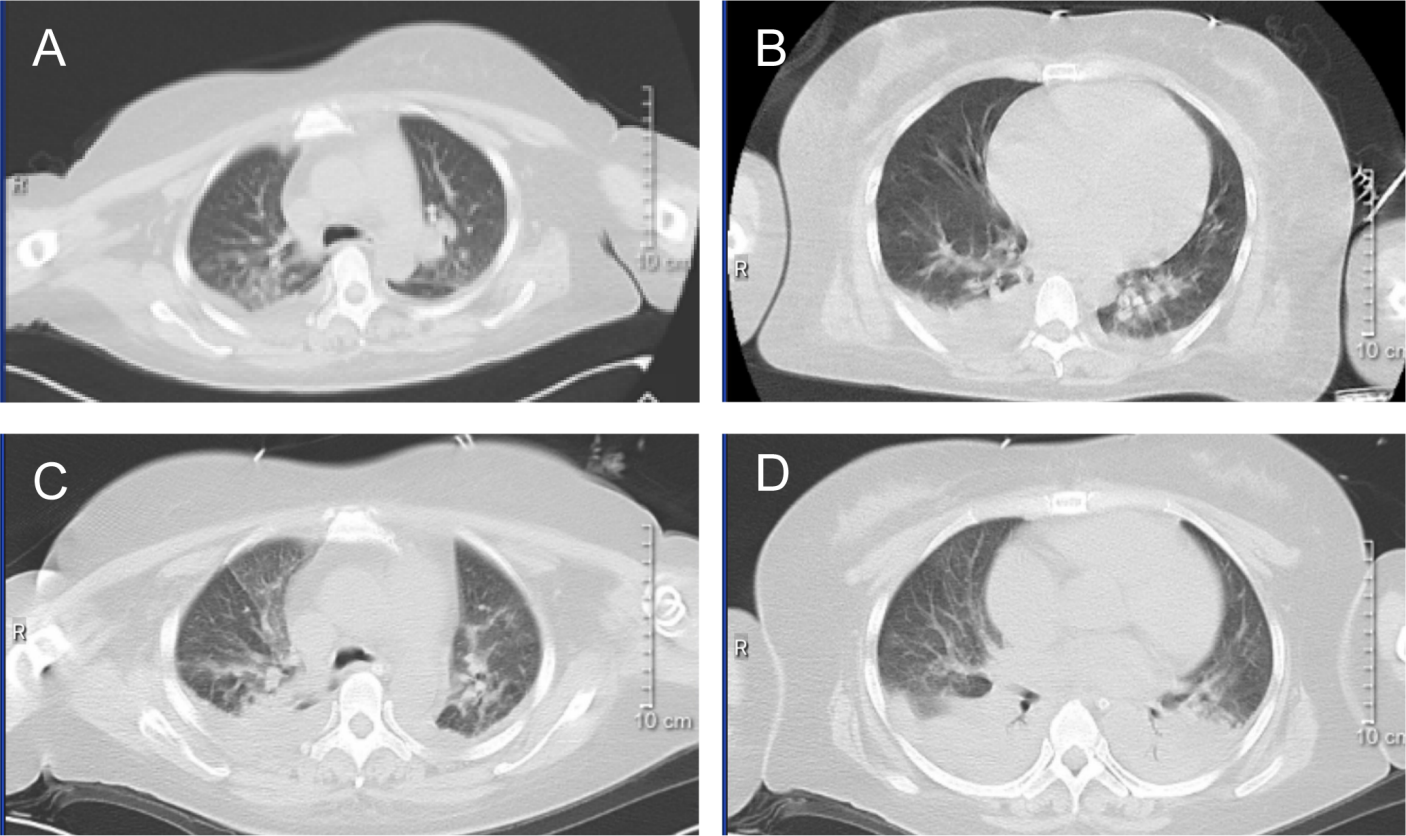
CT image. **(A, B)** the chest CT image of February 28, 2024. **(C, D)** the chest CT image of March 3, 2024.

**Figure 3:**
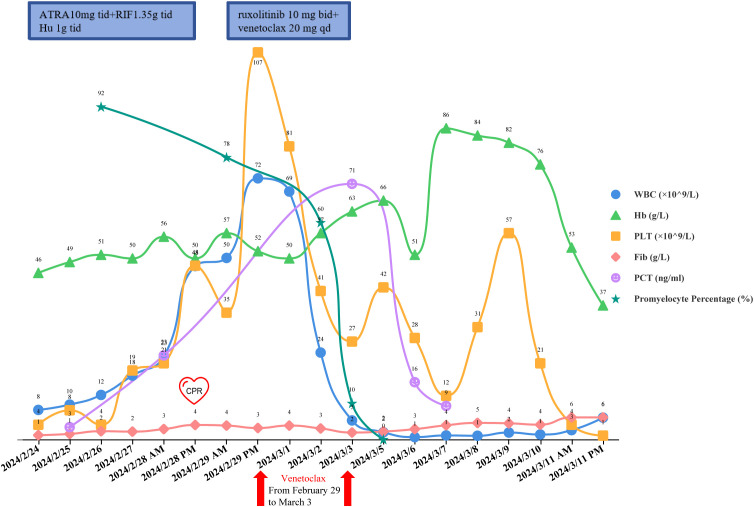
The changes of WBC, hemoglobin, platelets, fibrinogen, PCT, and the proportion of promyelocytes in the peripheral blood smear.

On February 26, the WBC were 12.24×10^9^/L, and on February 27, they increased to 17.59×10^9^/L. A rapid rise in leukocytes often signals the onset of DS. ATRA and RIF were immediately stopped. Given the patient’s lengthy history of schizophrenia, glucocorticoids may worsen the patient’s psychosis. Due to the patient’s lack of cooperation, the use of deep vein cannulation to administer conventional chemotherapy medications to achieve cytoreduction seemed to be extremely challenging. By 6:00 a.m. on February 28, laboratory tests revealed that WBC had increased to 22.91×10^9^/L and procalcitonin (PCT) was 23.17ng/ml. She also experienced dyspnea, chest distress, and palpitation, which prompted us to administer hydroxyurea 1g three times a day, meropenem for antibacterial therapy, and fluconazole as fungal prophylaxis. The Computed Tomography (CT) scan revealed pulmonary edema in both lungs, pulmonary infection, pleural effusion on both sides, and pericardial effusion (**Figures 2A, B**). The patient’s dyspnea worsened at 8:50 p.m. on February 28 and could not be alleviated by oxygen uptake. The patient unexpectedly experienced heart arrest and loss of consciousness at 21:00. Cardiopulmonary resuscitation (CPR) was done immediately by physicians and nurses, and the patient’s spontaneous heartbeat and respiration returned 5 minutes later, but her consciousness remained in a mild coma. The WBC was up to 47.89×10^9^/L, LDH 6250 U/L, plasma B-type natriuretic peptide (BNP) > 35000 pg/ml, Alanine Aminotransferase (ALT) 111 IU/L, and Aspartate Aminotransferase (AST) 299 IU/L. Cerebral bleeding was ruled out by CT scanning. For advanced life support, the patient was moved from the hematological ward to the intensive care unit (ICU). Given that hydroxyurea was ineffective in cytoreduction, we stopped it. We administered ruxolitinib 10 mg twice daily to ameliorate the cytokine storm syndrome and venetoclax 20 mg once daily for cytoreduction. On February 29, the WBC was 72.16×10^9^/L; on March 1, it was 68.51×10^9^/L; and on March 2, it fell to 23.96×10^9^/L. On March 3, it decreased to 5.19×10^9^/L, and venetoclax was suspended. The patient’s coagulation disorder was controlled during this period by receiving transfusions with fresh plasma, platelets, and red blood cells. After reaching a peak of 9181 U/L, LDH steadily dropped, ALT and AST returned to normal, and creatinine dropped from 573 μmol/L to 134.8 μmol/L. In the peripheral blood smear, the promyelocyte percentage dropped from 92% to 0. However, the PCT rose from the initial 3.34 ng/ml to 23.17 ng/ml and peaked at 70.63 ng/ml, and her temperature was consistently higher than 38°C. A novel hemorrhagic lesion marked by subarachnoid hemorrhage (SAH) was indicated by cranial CT. Bilateral pleural effusion and inflammation in the upper lobes of both lungs were revealed by chest CT (**Figures 2C, D**). Subcutaneous edema, abdominopelvic effusion, and perirenal exudate in both kidneys were indicated by abdominal CT. *Candida albicans* was discovered in the patient’s sputum cultures, whereas fungi were suggested by blood cultures. The patient was treated with meropenem, linezolid, voriconazole, and caspofungin. The patient’s temperature progressively dropped to 37.5°C, and the PCT fell from a high of 70.63 ng/ml to 9.21 ng/ml. SAH and bilateral pleural effusions were still suggested by CT on March 5. Laboratory test revealed WBC 1.84×10^9^/L, hemoglobin 82 g/L, platelets 57×10^9^/L, fibrinogen 4.39 g/L, creatinine 121 μmol/L, ALT 71 IU/L, and AST 41 IU/L on March 9, when the patient’s temperature had returned to normal. We restarted ATO 10 mg once daily and ATRA 10 mg three times daily. On March 11, the patient reemerged unexpectedly with a temperature of 39°C, hematochezia, and hematuria. Tests showed WBC 2.51×10^9^/L, hemoglobin 53 g/L, platelets 4×10^9^/L, fibrinogen 6.03 g/L, and creatinine 143.7 μmol/L. We suspended ATRA and ATO, administered ambulatory blood pressure monitoring, red blood cells and platelets transfusion, and treated the hematochezia and hematuria. On March 12, around 21:20, the cardiac monitor showed that the heart rate had decreased to 22 beats per minute and the blood pressure had dropped to 75/31 mmHg. The physicians and nurses performed CPR immediately, but the patient failed to regain spontaneous heartbeat and respiration and was declared clinically dead at 22:00. Two days later, the patient’s sputum culture revealed *Ralstonia mannitolilytica*, while the blood culture revealed *Enterococcus*. **Figure 3** displayed the changes of WBC, hemoglobin, platelets, fibrinogen, PCT, and the proportion of promyelocytes in the peripheral blood smear.

## Discussion

DS, a common adverse complication, frequently happens after the use of ATRA and ATO, and can be fatal in extreme circumstances (18–20). In 46% of patients, symptoms start in the first week, and in 38% of patients, they appear at the third week or later (21). Dyspnea, pulmonary infiltration, pleural and pericardial effusions, weight gain, hypotension, and renal failure are among the symptoms. As soon as DS is suspected, administer dexamethasone 10 mg/m^2^ immediately, once daily, or every 12 hours as needed (22). Once the signs and symptoms have subsided, which is typically within two weeks, dexamethasone should be tapered off. Depending on the degree of DS, discontinue the ATRA and/or ATO as necessary. Once symptoms subside, the therapeutic dose is gradually resumed. However, dexamethasone comes with a risk of inducing insanity (23). This may relate to the selective inhibition of spontaneous electrical activity, the development of neuronal hyperpolarization, and the enhanced involvement of receptor-like molecules on the cell membrane. The symptoms of insanity include euphoria, agitation, delirium, restlessness, disorientation, and depression. Patients with chronic wasting diseases and those with a history of schizophrenia are more likely to experience insanity. Administering dexamethasone in the rapidly developing DS is highly challenging given the patient’s 17-year history of taking ziprasidone, lorazepam, and clozapine. In patients with APL, DS frequently develops in tandem with a sharp rise in leukocytes. To lower early mortality in APL, the leukocyte rise must be quickly and efficiently controlled, and TLS needs to be prevented. The conventional cytoreduction therapeutic option is hydroxyurea because leukapheresis makes the patient’s coagulation condition worse. Nevertheless, this patient’s leukocytes were not significantly controlled by hydroxyurea. To avoid TLS during cytoreduction, sufficiently high-dose rehydration therapy is also necessary. Given the patient’s exceedingly poor cardiac function, the high-dose rehydration therapy may have worsened the heart failure and caused another cardiac arrest. Furthermore, when the administration of hydroxyurea failed to produce a cytoreductive effect, the chemotherapeutic drugs, such as anthracyclines, would be added right away. Cardiotoxicity is well known as the side effect of anthracyclines (24). However, the patient’s heart’s tolerance of anthracyclines was in doubt because she had pericardial effusion, the cardiac EF of just 44%, and myocardial ischemia when she was admitted.

When DS first started, this young female patient encountered numerous therapeutic conundrums. It appeared that this problem could not be solved by any of the conventional therapies. There are limited reports of cardiotoxicity and psychosis from venetoclax. We administered venetoclax 20 mg once daily as a cytoreduction therapy. As anticipated, the WBC dropped from a peak of 72.16×10^9^/L to 5.19×10^9^/L in 4 days, and the proportion of promyelocytes in the peripheral blood smear decreased from 78% to 10%. TLS did not develop since the patient received good supportive treatment in the ICU. Regretfully, due to severe infection and sudden cardiac arrest, the patient remained in a deep coma. She eventually died of sepsis and infectious shock.

There are limited studies on venetoclax as cytoreduction therapy in newly diagnosed adult patients with APL, and current studies mainly focus on pediatric APL. Results from a single-center study at Beijing Children’s Hospital showed that venetoclax as a cytoreduction therapy (20–50 mg/m^2^/d) for pediatric APL had a reduced incidence of disease-related complications, making it a superior option (11). The dose of venetoclax used in this female was 20 mg/d (for a total of 4 days, from February 29, 2024, to March 3, 2024). According to the venetoclax usage instructions, when used in combination with strong CYP3A inhibitors (such as itraconazole, voriconazole, ketoconazole, etc.), venetoclax should be reduced to 100 mg/day. If in the dose escalation phase, the reduction method is as follows: venetoclax 10 mg on day 1, 20 mg on day 2, 50 mg on day 3, and 100 mg on day 4. Since the patient received voriconazole treatment, and full-dose venetoclax could potentially induce TLS, we opted for a low-dose regimen. Fortunately, the patient’s WBC decreased steadily without developing TLS. Regrettably, no blood concentration monitoring of venetoclax was performed at that time. Regarding the effect of anti-schizophrenic medications on venetoclax, there is currently insufficient medical evidence and related research. Although anti-schizophrenic medications were discontinued after the patient was transferred to the ICU, further research is needed to confirm whether residual anti-schizophrenic medications in the blood affect venetoclax.

It is undeniable that venetoclax plays a critical role in cytoreduction in the early stages of DS, even though the patient’s final clinical outcome was death from sepsis. High-risk patients may benefit from this treatment approach, particularly those with heart failure who have trouble with traditional chemotherapeutic drugs such as anthracyclines. It is still necessary to investigate the venetoclax dose in newly diagnosed APL patients, as well as drug concentration monitoring and TLS prevention.

## Conclusions

For newly diagnosed adult patients with APL, venetoclax may work well as a cytoreduction therapy option, particularly for those who have not responded well to or are intolerant of traditional cytoreduction treatment. To determine the optimal therapeutic dosage and the long-term safety and efficacy of venetoclax in APL, more research is needed.

## Data Availability

The original contributions presented in the study are included in the article/supplementary material. Further inquiries can be directed to the corresponding author.
